# Protective effect of smoking cessation on subsequent myocardial infarction and ischemic stroke independent of weight gain: A nationwide cohort study

**DOI:** 10.1371/journal.pone.0235276

**Published:** 2020-07-16

**Authors:** Jung-Hwan Cho, Hye-Mi Kwon, Se-Eun Park, Jin-Hyung Jung, Kyung-Do Han, Yong-Gyu Park, Yang-Hyun Kim, Eun-Jung Rhee, Won-Young Lee

**Affiliations:** 1 Division of Endocrinology and Metabolism, Department of Internal Medicine, Kangbuk Samsung Hospital, Sungkyunkwan University School of Medicine, Seoul, Republic of Korea; 2 Department of Biostatistics, Biomedicine & Health Sciences, Catholic University College of Medicine, Seoul, Republic of Korea; 3 Department of Family Medicine, Korea University Hospital, College of Medicine, Korea University, Seoul, Republic of Korea; Medical University of South Carolina, UNITED STATES

## Abstract

Smoking cessation reduces the cardiovascular risk but increases body weight. We investigated the risk of subsequent myocardial infarction and ischemic stroke according to weight gain after smoking cessation, using a nationwide population based cohort. We enrolled 3,797,572 Korean adults aged over 40 years who participated in national health screenings between 2009 and 2010. Subjects who quit smoking were classified into three subgroups according to the weight change between baseline and 4 years prior. Myocardial infarctions and ischemic strokes were followed until the end of 2015. We compared the hazard ratios among smoking cessation subgroups, non-smokers, and current smokers. The mean changes in weight (1.5 ± 3.9 kg) of the smoking cessation group were higher than those of the other groups (p < 0.0001). A total of 31,277 and 46,811 subjects were newly diagnosed with myocardial infarction and ischemic stroke, respectively. Regardless of weight change, all subgroups of smoking cessation had significantly less risk than current smokers. The subgroup of smoking cessation with weight gain over 4kg showed the lowest risk for myocardial infarctions (hazard ratio 0.646, 95% confidence interval 0.583–0.714, p < 0.0001) and ischemic strokes (hazard ratio 0.648, 95% confidence interval 0.591–0.71, p < 0.0001) after multivariable adjustment. In conclusion, weight gain after smoking cessation did not adversely affect the cardiovascular protective effect.

## Introduction

Smoking is an important, absolute risk factor in health behavior that can be corrected and significantly affects the incidence and mortality of cardiovascular disease (CVD) [1]. Since smoking is known to be related to the onset of inflammatory responses and the progression of thrombosis through increased oxidative stress and endothelial dysfunction, smoking cessation prevents the progression of atherothrombosis by rapidly reversing hemostatic and inflammatory markers [2]. However, people who quit smoking were exposed to the risk of weight gain resulting from smoking cessation [3]. Smoking cessation increases food intake and decreases energy expenditure via the effects of nicotine deficiency [4]. As the energy balance moves in a positive direction, obesity becomes worse and the metabolic profiles deteriorate, increasing the risk factors for cardiovascular disease including metabolic syndromes [5, 6].

Several studies investigated how weight gain after smoking cessation affect the relative risk of CVD. In the study of Japanese male [7], successful abstainers had an average weight gain of 2.4 kg over the 4 years after smoking cessation. Blood pressure, total cholesterol, triglyceride and fasting blood glucose of these subjects were also significantly worsened. Nevertheless, the estimated risk of CVD was reduced by 24% as compared to the baseline, conversely increased by 9% in continuing smokers. In the study using cohort data from the Framingham Offspring Study [8], weight gain following smoking cessation did not modify the association between quitting and lowering risk of CVD even though it did not yield enough statistical power in diabetic subjects. Subsequently, a study conducted in Korea with more participants demonstrated that quitters with change in BMI greater than 1.0 kg/m2 decreased the risk of myocardial infarction by 67%, and total stroke by 25% smokers with statistical significance compared to sustained smokers after adjusting for cardiovascular risk factors including blood glucose [9]. However, this study was only included males over 40 years of age because of the few number of female smokers. In addition, the body mass index (BMI) used to represent weight change in this study is affected by an individual's height. In a recent longitudinal cohort study performed in the United States [10], smoking cessation was associated with an increased short-term risk of type 2 diabetes with substantial weight gain over 5kg, but consistently reduced all-cause and cardiovascular mortality during extended follow-up durations regardless of the degree of weight gain in both men and women. However, recent quitters that did not gain weight had a higher risk of mortality relative to those who gained weight. Cardiovascular prognosis and mortality seems better in overweight or obese described by the “obesity paradox” [11], so the risk of actual cardiovascular events due to weight gain might be underestimated when determining mortality as an outcome.

Therefore, we aimed to investigate how the real world incidences and the risk of subsequent myocardial infarction (MI) and ischemic stroke (IS) were related to the weight gain after smoking cessation in nationwide Korean adults, through utilizing the national data of health screenings and insurance records.

## Materials and methods

### Study population

More than 95% of Koreans are covered by the National Health Insurance Service (NHIS). Nearly all Korean adults over 40 years of age undergo regular health checkups provided by the NHIS every one or two years. The NHIS organizes health checkup data together with national insurance claim records to provide comprehensive information for medical research from patient demographic information to hospital records. This information includes examinations, laboratory findings, diagnoses, and treatments. This database contains a representative population based cohort widely applicable to various clinical studies [12]. Our study was approved by the NHIS (Research Number: NHIS-2019-1-483).

**Fig 1:**
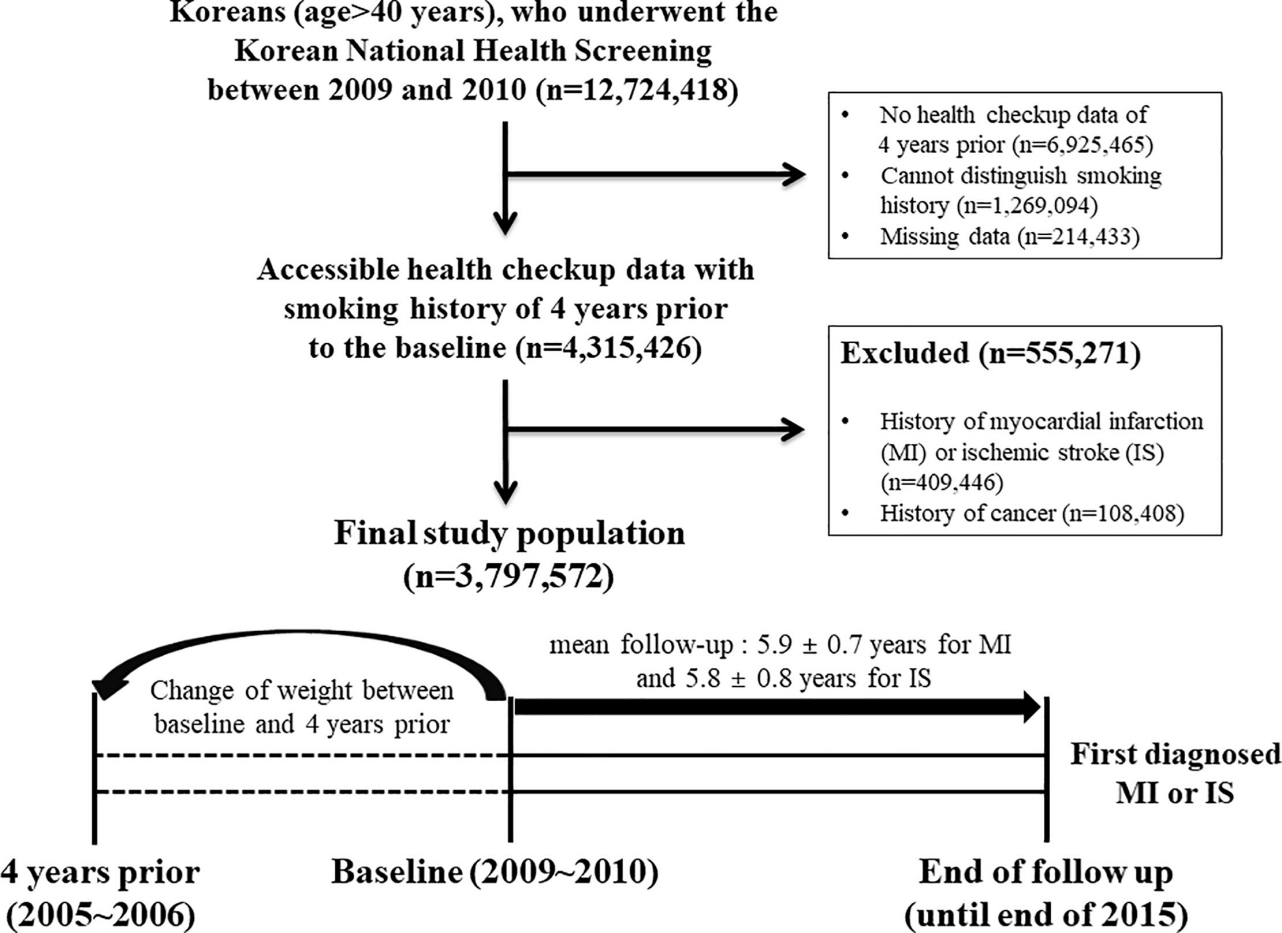
The selection process of study population.

A total of 12,724,418 Korean adults over 40 years of age participated in national health screenings between 2009 and 2010. Of these, 4,315,426 people who did not have missing data and whose health screening data including smoking history from 4 years prior could be accessed were included in this study. Individuals diagnosed with previous MIs or ISs (409,446) were excluded, as were 108,408 individuals previously diagnosed with cancer due to the possibility of unintentional weight change. Ultimately, we enrolled 3,797,572 subjects (Fig 1).

### Definition of smoking history and outcomes

We evaluated the subjects’ smoking history through self-questionnaires during health screenings including their current smoking status, duration, and amount of smoking. The participants responded to their current smoking status through one of three choices: never smoked, smoking in the past but now quitting, or continuing to smoke. Current smokers were defined as those who responded to smoke continuously from 4 years prior to baseline. Those who consistently never smoked were classified as non-smokers. The participants who quit smoking at baseline, but who were smokers 4 years prior were classified as the smoking cessation group.

Weight change was calculated as the difference of weight between baseline and 4 years prior. We reviewed all the participants’ international classification of disease (ICD) code claims through NHIS until the end of 2015 to verify the MI or IS diagnoses. The occurrence of a MI was defined as an ICD-10 I21 or I22 code claimed at least twice, or more than once with a hospitalization. The occurrence of an IS was defined as an ICD-10 I63 or I64 code claimed together with a hospitalization and a radiological examination (magnetic resonance imaging or computed tomography). The participants who had a history of MI or IS identified using these ICD code claims prior to baseline were excluded. Newly diagnosed MIs and ISs were followed from enrolment to the end of 2015. the mean follow-up periods for MI and IS were 5.9 ± 0.7 years and 5.8 ± 0.8 years, respectively.

### Other baseline characteristics

This study included both male and female Koreans aged over 40 years. Socio-behavioral information such as drinking (more than 30 g of alcohol per day), regular physical activity (moderate exercise more than 3 days per week or vigorous exercise more than 3 days per week), and income (below the 20th percentile) were obtained through a standardized questionnaire. Weight (kg) was measured with an electronic scale and BMI was calculated using height (cm) and body weight. Waist circumference (cm) was measured by trained examiners at the midpoint between the rib cage and iliac crest. Abdominal obesity was defined in accordance with the standard criteria in Korean male and female (≥90 and ≥85 cm, respectively) [13]. Blood samples were collected during fasting and blood pressure was measured by a skilled examiner using a sphygmomanometer after a five minute rest. Hypertension, diabetes, and hyperlipidemia were diagnosed using previous claim record ICD codes (ICD-10 code I10 to I15; E11 to 14; E78) with medication or checking results from health screenings (systolic blood pressure ≥ 140 mmHg and diastolic blood pressure ≥ 90 mmHg; fasting blood glucose level ≥ 126 mg/dl; total cholesterol levels ≥ 240 mg/dl).

### Statistical analysis

In order to demonstrate the effectiveness of smoking cessation, we performed primary analysis by identifying the crude incidence rate (IR) and the hazard ratios (HR) of MI or IS. HRs were compared among the smoking cessation, non-smoker, and current-smoker groups with current smokers as a reference group. We analyzed HR by performing multivariate adjustments with confounders from a non-adjusted model to a fully adjusted model (Model 1, non-adjusted; Model 2, age and sex; Model 3, age, sex, and baseline BMI; Model 4, age, sex, baseline BMI, alcohol drinking, low income, and regular exercise). To demonstrate the effects of weight change on the outcome, the smoking cessation group was divided into three subgroups based on cut-offs related to weight changes (≤ 0; 0–4; ≥ 4) and tertiles, and HRs of each divided subgroups were compared with current smokers and non-smokers. In addition, we performed secondary subgroup analyses to identify whether differences in sex, age, and the presence or absence of metabolic diseases such as hypertension, diabetes, hyperlipidemia, or abdominal obesity.

HRs in primary and secondary analyses were analyzed using a Cox proportional hazards model with a 95% confidence interval (CI). Continuous variables were analyzed by variance analysis, and categorical variables were analyzed using the chi-squared test. Statistical calculations and analyses were performed using SAS version 9.3 (SAS Institute Inc., Cary, NC, USA). Since the cohort data of NHIS was anonymous and adhered to confidentiality guidelines, we were exempt from securing informed consent. Our research complied with the Declaration of Helsinki through approved by the official review committee and the institutional review board of the Kangbuk Samsung Hospital (IRB Number: KBSMC 2017-06-004).

## Results

### Baseline characteristics

Of the total 3,797,572 subjects, approximately 4.5% quit smoking (n = 172,439) and 20% continued to smoke (n = 768,087) (Table 1). Smoking rates were higher in males than females and the amount of smoking in past smokers was about 21 pack-years. About 61% of the subjects who quit smoking gained weight. The mean weight change of the total smoking cessation group at baseline was 1.5 ± 3.9 kg, which was 0 ± 3.3 kg for non-smokers and 0 ± 3.6 kg for current smokers (p <0.0001). In addition to weight gain, systolic (1.0 ± 16.1 mmHg) and diastolic blood pressure (0.3 ± 11.5 mmHg), blood cholesterol (5.5 ± 34.7 mg/dl), and fasting blood sugar (5.1 ± 26.0 mg/dl) increased in the smoking cessation group at baseline compared to the 4 year prior.

**Table 1:** Baseline characteristics of the subjects according to smoking status.

	Non-smoker	Smoking cessation	Current smoker
N (%)	2,857,046 (75.23)	172,439 (4.54)	768,087 (20.23)
Sex			
Male, N (%)	704,700 (24.67)	168,107 (97.49)	744,941 (96.99)
Female, N (%)	2,152,346 (75.33)‬	4,332 (2.51)	23,146 (3.01)
Amount of smoking, pack-years, Mean (SD)	0	21.2 ± 16.1	22.2 ± 13.7
0–10, N (%)		35,657 (20.68)	103,939 (13.53)
10–20, N (%)		49,385 (28.64)	221,667 (28.86)
≥20, N (%)		87,397 (50.68)	442,481 (57.61)
Age (group), Mean (SD)	55.8 ± 9.9	52.32 ± 9.2	51.5 ± 9.1
≥55, N (%)	1,369,268 (47.93)	59,023 (34.23)	234,855 (30.58)
Alcohol drinking^a^, N (%)	55,318 (1.94)	25,732 (14.92)	136,818 (17.81)
Low income^b^, N (%)	559,664 (19.59)	22,481 (13.04)	111,680 (14.54)
Regular exercise^c^, N (%)	592,791 (20.75)	46,173 (26.78)	149,136 (19.42)
Hypertension, N (%)	902,467 (31.59)	56,810 (32.94)	219,192 (28.54)
Diabetes, N (%)	267,412 (9.36)	22,669 (13.15)	95,159 (12.39)
Hyperlipidemia, N (%)	685,405 (23.99)	40,664 (23.58)	151,655 (19.74)
Abdominal obesity^d^, N (%)	583,814 (20.43)	45,048 (26.12)	161,166 (20.98)
Waist circumference (cm), Mean (SD)	79.4 ± 8.4	85.0 ± 7.3	83.5 ± 7.6
Body mass index, Mean (SD)	23.8 ± 3.0	24.5 ± 2.8	23.8 ± 2.9
Height (cm), Mean (SD)	158.2 ± 8.0	168.7 ± 6.2	168.5 ± 6.4
Weight (kg), Mean (SD)	59.8 ± 9.5	69.9 ± 9.8	67.8 ± 10.2
Weight difference (subgroup), Mean (SD)	0 ± 3.3	1.5 ± 3.9	0 ± 3.6
≤ 0, N (%)	1,612,958 (56.46)	67,387 (39.08)	433,313 (56.41)
0–4, N (%)	922,931 (32.3)	57,799 (33.52)	233,034 (30.34)
≥ 4, N (%)	321,157 (11.24)	47,253 (27.4)	101,740 (13.25)
Systolic blood pressure (mmHg), Mean (SD)	123.1 ± 15.3	125.6 ± 14.1	124.4 ± 14.3
Difference, Mean (SD)	-0.1 ± 16.5	1.0 ± 16.1	-0.4 ± 16.0
Diastolic blood pressure (mmHg), Mean (SD)	76.2 ± 9.9	78.9 ± 9.7	78.2 ± 9.8
Difference, Mean (SD)	-0.5 ± 11.3	0.3 ± 11.5	-0.6 ± 11.4
Fasting plasma glucose (mg/dl), Mean (SD)	97.6 ± 21.0	102.7 ± 26.3	101.6 ± 27.1
Difference, Mean (SD)	3.1 ± 21.0	5.1 ± 26.0	3.9 ± 27.0
Total cholesterol (mg/dl), Mean (SD)	201.3 ± 36.6	202.0 ± 36.5	199.2 ± 36.2
Difference, Mean (SD)	4.2 ± 35.3	5.5 ± 34.7	2.6 ± 33.2
Prevalence of myocardial infarction, N (%)	20,321 (0.71)	1,613 (0.94)	9,343 (1.22)
Prevalence of ischemic stroke, N (%)	33,087 (1.16)	2,005 (1.16)	11,719 (1.53)

All characteristics met p < 0.0001.

^a^Defined as drinking more than 30 g of alcohol per day.

^b^Defined as moderate exercise more than 3 days per week or vigorous exercise more than 3 days per week.

^c^Defined as income below the 20^th^ percentile.

^d^Defined as waist circumference ≥ 90 cm in male and ≥ 85 cm in female.

### Effect of smoking cessation on the outcome

A total of 31,277 subjects (0.82%) were diagnosed with MIs and 46,811 subjects (1.23%) were diagnosed with ISs. The crude incidence of MI (IR 1.603 per 1,000 person-years) and IS (IR 1.994 per 1,000 person-years) were higher in the smoking cessation group than in non-smokers, but lower than that in current smokers. After multivariable adjustment, smoking cessation significantly lowered the risk of MIs (Model 4; HR 0.703, 95% CI 0.666–0.741, p < 0.0001) compared to current smokers. In the analysis of IS, smoking cessation consistently lowered the risk (Model 4; HR 0.7, 95% CI 0.667–0.734, p < 0.0001) (Table 2).

**Table 2:** Incidence Rate (IR) and multivariate-adjusted Hazard Ratios (HRs) (95% confidence intervals) of myocardial infarction and ischemic stroke.

Myocardial infarction							
				HR (95% Cl)			
Smoking status	Number of subjects	Events	IR (per 1000 person years)	Model 1^a^	Model 2^b^	Model 3^c^	Model 4^d^
Non-smoker	2,857,046	20,321	1.215	0.582 (0.567–0.596)	0.485 (0.47–0.501)	0.473 (0.458–0.488)	0.47 (0.455–0.485)
Smoking cessation	172,439	1,613	1.603	0.768 (0.728–0.809)	0.724 (0.686–0.763)	0.7 (0.663–0.738)	0.703 (0.666–0.741)
Current smoker	768,087	9,343	2.088	1 (Ref.)	1 (Ref.)	1 (Ref.)	1 (Ref.)
Ischemic stroke							
				HR (95% Cl)			
Smoking status	Number of subjects	Events	IR (per 1000 person years)	Model 1^a^	Model 2^b^	Model 3^c^	Model 4^d^
Non-smoker	2,857,046	33,087	1.982	0.755 (0.739–0.771)	0.563 (0.548–0.578)	0.55 (0.536–0.565)	0.564 (0.55–0.58)
Smoking cessation	172,439	2,005	1.994	0.76 (0.725–0.797)	0.706 (0.673–0.74)	0.689 (0.656–0.722)	0.7 (0.667–0.734)
Current smoker	768,087	11,719	2.623	1 (Ref.)	1 (Ref.)	1 (Ref.)	1 (Ref.)

^a^Non-adjusted.

^b^Adjusted for age and sex.

^c^Adjusted for age, sex, and body mass index.

^d^Adjusted for age, sex, body mass index, alcohol drinking, low income, and regular exercise.

### Difference in the effect according to the change in weight

Regardless of changes in weight, all smoking cessation subgroups showed significantly less risk than current smokers when spanning from the non-adjusted model to the fully adjusted model (Table 3). The subgroup of smoking cessation with weight gain over 4kg (mean increase in weight: 6.0 ± 2.4 kg) showed the lowest risk for MIs (Model 4; HR 0.646, 95% CI 0.583–0.714, p < 0.0001) and ISs (Model 4; HR 0.648, 95% CI 0.591–0.71, p < 0.0001).

**Table 3:** Incidence Rate (IR) and multivariate-adjusted Hazard Ratios (HRs) (95% confidence intervals) of myocardial infarction and ischemic stroke according to the change in weight.

Myocardial infarction							
				HR (95% Cl)			
Smoking status (weight change)	Number of subjects	Events	IR (per 1000 person years)	Model 1^a^	Model 2^b^	Model 3^c^	Model 4^d^
Non-smoker	2,857,046	20,321	1.215	0.582 (0.567–0.596)	0.485 (0.47–0.501)	0.473 (0.458–0.488)	0.47 (0.455–0.485)
Smoking cessation							
≤ 0	67,387	722	1.845	0.884 (0.819–0.953)	0.78 (0.723–0.841)	0.778 (0.721–0.839)	0.782 (0.725–0.843)
0–4	57,799	499	1.477	0.707 (0.646–0.773)	0.674 (0.615–0.737)	0.648 (0.592–0.708)	0.651 (0.594–0.712)
≥ 4	47,253	392	1.416	0.678 (0.612–0.749)	0.696 (0.628–0.769)	0.645 (0.582–0.712)	0.646 (0.583–0.714)
Current smoker	768,087	9,343	2.088	1 (Ref.)	1 (Ref.)	1 (Ref.)	1 (Ref.)
Ischemic stroke							
				HR (95% Cl)			
Smoking status (weight change)	Number of subjects	Events	IR (per 1000 person years)	Model 1^a^	Model 2^b^	Model 3^c^	Model 4^d^
Non-smoker	2,857,046	33,087	1.982	0.755 (0.739–0.771)	0.563 (0.548–0.578)	0.55 (0.536–0.565)	0.565 (0.55–0.58)
Smoking cessation							
≤ 0	67,387	915	2.340	0.893 (0.834–0.954)	0.758 (0.709–0.811)	0.757 (0.707–0.809)	0.772 (0.721–0.825)
0–4	57,799	617	1.828	0.697 (0.642–0.755)	0.658 (0.607–0.713)	0.639 (0.589–0.692)	0.649 (0.598–0.704)
≥ 4	47,253	473	1.710	0.651 (0.594–0.713)	0.68 (0.619–0.744)	0.642 (0.584–0.703)	0.648 (0.591–0.71)
Current smoker	768,087	11,719	2.623	1 (Ref.)	1 (Ref.)	1 (Ref.)	1 (Ref.)

The change in weight was calculated as the weight difference between baseline and four years prior.

The mean ± standard deviation increase in weight (kg) was -2.1 ± 2.5 at ≤ 0; 2.0 ± 0.8 at 0–4; 6.0 ± 2.4 at ≥ 4.

^a^Non-adjusted.

^b^Adjusted for age and sex,.

^c^Adjusted for age, sex, and body mass index.

^d^Adjusted for age, sex, body mass index, alcohol drinking, low income, and regular exercise.

When the subgroups were divided into tertiles, the 3rd tertile (mean increase in weight: 5.2 ± 2.4 kg) showed the lowest risk for MIs (HR 0.641, 95% CI 0.587–0.699, p < 0.0001), and the 2nd tertile (mean increase in weight: 1.0 ± 0.8 kg) showed the lowest risk for ISs (HR 0.657, 95% CI 0.606–0.711, p < 0.0001) (S1 Table). In secondary subgroup analyses, the reduction of risk after smoking cessation was consistent across the categories of sex, age, hypertension, diabetes, hyperlipidemia, and abdominal obesity (S2 Table).

## Discussion

According to our retrospective cohort study of about 3.8 million nationwide Korean adults, weight gained on average and metabolic profiles were worsened after smoking cessation. Before our study, numerous studies have confirmed increases in weight gain after quitting [7, 14, 15]. Smoking increases insulin resistance and central fat accumulation, raising the risk of metabolic syndromes and diabetes [16]. In the early stages of smoking cessation, continuous increased β-cell secretion in response to glucose and fasting insulin resistance is responsible for weight gain, along with an increase in energy balance due to nicotine withdrawal [17]. Several studies have shown an increase in visceral adipose after quitting, which over time gradually dropped compared to that of non-smokers [6, 18]. Changes in abdominal obesity, insulin resistance, and the incidence of type 2 diabetes mellitus showed a similar pattern that increases after smoking cessation and then decreases [10, 19].

Despite weight gain, we found that the risk of MIs and ISs in quitters was lower than current smokers. Furthermore, weight gain after quitting was not associated with a relative increase in the risk. Several mechanisms could explain the prevention of cardiovascular disease despite weight gain and the deteriorated metabolic profile after smoking cessation. Post-cessation-related obesity could contribute to insulin resistance, but the benefits of stopping smoking due to reverse the worsening of insulin resistance caused by nicotine outweigh the risks [20]. Insulin modulates lipoprotein lipase (LPL) activity, and this enzyme expressed by the adipose tissue has an anti-atherogenic effect through improving circulating lipoprotein profiles [21]. The paradoxical response of adipose LPL to glucose in smokers was improved by stopping smoking, contributing to weight gain and increasing adipose tissue LPL activity [22, 23]. Researchers from the REGRESS study group demonstrated that LPL activity was inversely associated with severity of angina pectoris [24]. A clinical study investigating the preheparin LPL mass in patients with coronary atherosclerosis showed that the amount of preheparin serum LPL was significantly lower in patients with coronary atherosclerosis, which was negatively correlated with triglycerides and positively correlated with HDL cholesterol [25]. Smoking is a more potent mediator for normalization of cardio-protective high density lipoprotein cholesterol (HDL-C) than weight gain [26]. In aforementioned study of Japanese male, it was observed that HDL-C increased steadily despite weight gain after smoking cessation [7]. Adiponectin, which is a fundamental factor of lipid metabolism and plays an important role in the development of cardiovascular disease, increases after smoking cessation and does not decrease in spite of increased body weight and abdominal obesity due to quit smoking [27].

One interesting result which emerged from our study was that the group with weight loss after smoking cessation had a significantly lower risk of MIs or ISs than current smokers, but seemed to have a higher incidence and relative risk compared to the group with weight gain after quitting. In the previous study of Koreans with similar designs to ours, no significant risk reduction of MI and total stroke were identified in quitters with BMI loss compared to sustained smokers [9]. This tendency to increase the relative risk of the weight loss group might have been influenced by other causes accompanying unintentional weight loss and/or sarcopenia, despite we excluded patients previously diagnosed with cancer. Subsequent unintentional weight loss due to aging, health problems or chronic disease is closely related to sarcopenia [28]. Several studies in Koreans found the association between sarcopenia and the risk of cardiovascular disease [29, 30]. In addition, we did not exactly know why the subjects of our study quit smoking and there is a possibility that those who attempted to quitting were influenced by other health problems. Therefore, it would be necessary to conduct further research that including the causes and intentions of weight loss after smoking cessation, and whether accompanied by sarcopenia or not.

Our study including both sexes and all adults over 40 years of age who had undergone periodic medical examinations across the country, represented the entire nation of Korean adults. We used the measured weight rather than self-checked weight to reduce error. In order to ensure statistical significance, large number of individuals in the smoking cessation group were included, and various models were calibrated to minimize confounding outcomes between smoking cessation and weight change. Despite these efforts, our study had several limitations. First, our study only analyzed one-time point for smoking cessation and we could not confirm the exact starting point and duration of smoking cessation. This ‘point prevalence’ design which could not identify the changes of exposure over time could lead to an error in weight gain measurement and result interpretation [10, 31]. Secondly, a selection bias cannot be ruled out because only the participants with access to the medical examination record and smoking history of the 4 years prior were allowed to the study. Thirdly, there is a possibility of bias in the confirmation of characteristics through self-questionnaires and the diagnosis of outcomes through insurance claims. Lastly, since we included only Koreans in our study, the effect of smoking cessation on weight gain and its impact on the outcome may be different in other countries and ethnicities.

In conclusion, smoking cessation was effective in reducing the risk of MIs and ISs. Although weight gain after smoking cessation had a temporary adverse effect on the metabolic profile, it did not affect the protection that smoking cessation provided to the cardiovascular system.

## Supporting information

S1 Table Incidence Rate (IR) and multivariate adjusted Hazard Ratios (HRs) (95% confidence intervals) of myocardial infarctions and ischemic strokes according to the tertiles of weight change. (DOCX)

S2 Table Secondary subgroup analyses according to sex, age, and the presence or absence of hypertension, diabetes, hyperlipidemia, or abdominal obesity. (DOCX)
