# Supplementary material for: Protective effect of smoking cessation on subsequent myocardial infarction and ischemic stroke independent of weight gain: A nationwide cohort study
Source: PLoS One. 2020 Jul 16;15(7):e0235276. doi: 10.1371/journal.pone.0235276 (PMC7365437; doi:10.1371/journal.pone.0235276)
Supplement: S1 Table — (DOCX) [file pone.0235276.s001.docx]

**S1 Table**. **Incidence rate (IR) and multivariate-adjusted Hazard Ratios (HRs) (95% confidence intervals) of myocardial infarction and ischemic stroke according to the tertiles of weight change.**

Myocardial infarction

|  |  |  |  | HR (95% Cl) | | | |
| --- | --- | --- | --- | --- | --- | --- | --- |
| Smoking status  (weight change) | Number of subjects | Events | IR (per 1000 person years) | Model 1^a^ | Model 2^b^ | Model 3^c^ | Model 4^d^ |
| Non-smoker | 2,857,046 | 20,321 | 1.215 | 0.582 (0.567–0.596) | 0.485 (0.47–0.501) | 0.473 (0.458–0.488) | 0.47 (0.455–0.485) |
| Smoking cessation |  |  |  |  |  |  |  |
| T1 | 48,622 | 540 | 1.917 | 0.919 (0.842–1.001) | 0.798 (0.731–0.869) | 0.801 (0.734–0.873) | 0.805 (0.738–0.877) |
| T2 | 58,912 | 536 | 1.557 | 0.746 (0.683–0.812) | 0.697 (0.639–0.76) | 0.678 (0.62–0.738) | 0.681 (0.623–0.742) |
| T3 | 64,905 | 537 | 1.413 | 0.677 (0.619–0.737) | 0.685 (0.627–0.747) | 0.639 (0.585–0.696) | 0.641 (0.587–0.699) |
| Current smoker | 768,087 | 9,343 | 2.088 | 1 (Ref.) | 1 (Ref.) | 1 (Ref.) | 1 (Ref.) |

Ischemic stroke

|  |  |  |  | HR (95% Cl) | | | |
| --- | --- | --- | --- | --- | --- | --- | --- |
| Smoking status  (weight change) | Number of subjects | Events | IR (per 1000 person years) | Model 1^a^ | Model 2^b^ | Model 3^c^ | Model 4^d^ |
| Non-smoker | 2,857,046 | 33,087 | 1.982 | 0.755 (0.739–0.771) | 0.563 (0.548–0.578) | 0.55 (0.536–0.565) | 0.565 (0.55–0.58) |
| Smoking cessation |  |  |  |  |  |  |  |
| T1 | 48,622 | 690 | 2.451 | 0.936 (0.866–1.01) | 0.777 (0.719–0.838) | 0.779 (0.721–0.84) | 0.794 (0.735–0.857) |
| T2 | 58,912 | 646 | 1.877 | 0.716 (0.661–0.774) | 0.66 (0.609–0.714) | 0.645 (0.596–0.698) | 0.657 (0.606–0.711) |
| T3 | 64,905 | 669 | 1.762 | 0.671 (0.621–0.725) | 0.688 (0.636–0.743) | 0.653 (0.603–0.705) | 0.66 (0.61–0.713) |
| Current smoker | 768,087 | 11,719 | 2.623 | 1 (Ref.) | 1 (Ref.) | 1 (Ref.) | 1 (Ref.) |

The change in weight was calculated as the weight difference between baseline and four years prior.

The mean + standard deviation increase in weight (kg) was -3.0 ± 2.4 at T1; 1.0 ± 0.8 at T2; 5.2 ± 2.4 at T3.

^a^Non-adjusted.

^b^Adjusted for age, sex, and body mass index.

^c^Adjusted for age, sex, body mass index, alcohol drinking, low income, and regular exercise.

^d^Adjusted for age, sex, body mass index, alcohol drinking, low income, regular exercise, hypertension, diabetes, and hyperlipidemia.
