# Supplementary material for: Protective effect of smoking cessation on subsequent myocardial infarction and ischemic stroke independent of weight gain: A nationwide cohort study
Source: PLoS One. 2020 Jul 16;15(7):e0235276. doi: 10.1371/journal.pone.0235276 (PMC7365437; doi:10.1371/journal.pone.0235276)
Supplement: S2 Table — (DOCX) [file pone.0235276.s002.docx]

**S2 Table. Secondary subgroup analyses according to sex, age and presence or absence of hypertension, diabetes, hyperlipidemia or abdominal obesity.**

Myocardial infarction

| Subgroup | Smoking status  (weight change) | Number of subjects | Events | IR (per 1000 person years) | HR^a^ (95% Cl) | p-Interaction |
| --- | --- | --- | --- | --- | --- | --- |
| Sex |  |  |  |  |  | 0.7055 |
| Male | Non-smoker | 704,700 | 5,928 | 1.439 | 0.492 (0.476–0.51) |  |
|  | Smoking cessation |  |  |  |  |  |
|  | ≤ 0 | 65,508 | 701 | 1.842 | 0.792 (0.734–0.856) |  |
|  | 0–4 | 56,358 | 492 | 1.494 | 0.663 (0.605–0.726) |  |
|  | ≥ 4 | 46,241 | 383 | 1.413 | 0.645 (0.582–0.715) |  |
|  | Current smoker | 744,941 | 9,008 | 2.076 | 1 (Ref.) |  |
| Female | Non-smoker | 2,152,346 | 14,393 | 1.142 | 0.495 (0.444–0.552) |  |
|  | Smoking cessation |  |  |  |  |  |
|  | ≤ 0 | 1,879 | 21 | 1.937 | 0.87 (0.56–1.352) |  |
|  | 0–4 | 1,441 | 7 | 0.835 | 0.411 (0.195–0.87) |  |
|  | ≥ 4 | 1,012 | 9 | 1.536 | 0.716 (0.369–1.387) |  |
|  | Current smoker | 23,146 | 335 | 2.493 | 1 (Ref.) |  |
| Age |  |  |  |  |  | <0.0001 |
| Age < 55 | Non-smoker | 1,487,778 | 4,134 | 0.475 | 0.436 (0.411–0.462) |  |
|  | Smoking cessation |  |  |  |  |  |
|  | ≤ 0 | 42,089 | 237 | 0.958 | 0.674 (0.591–0.769) |  |
|  | 0–4 | 38,270 | 215 | 0.954 | 0.648 (0.565–0.743) |  |
|  | ≥ 4 | 33,057 | 167 | 0.857 | 0.559 (0.479–0.653) |  |
|  | Current smoker | 533,232 | 4,254 | 1.359 | 1 (Ref.) |  |
| Age ≥ 55 | Non-smoker | 1,369,268 | 16,187 | 2.019 | 0.513 (0.493–0.534) |  |
|  | Smoking cessation |  |  |  |  |  |
|  | ≤ 0 | 25,298 | 485 | 3.366 | 0.867 (0.79–0.952) |  |
|  | 0–4 | 19,529 | 284 | 2.524 | 0.662 (0.587–0.746) |  |
|  | ≥ 4 | 14,196 | 225 | 2.743 | 0.717 (0.628–0.82) |  |
|  | Current smoker | 234,855 | 5,089 | 3.785 | 1 (Ref.) |  |
| Hypertension |  |  |  |  |  | 0.0035 |
| No | Non-smoker | 1,954,579 | 9,274 | 0.810 | 0.447 (0.427–0.468) |  |
|  | Smoking cessation |  |  |  |  |  |
|  | ≤ 0 | 45,259 | 368 | 1.394 | 0.761 (0.684–0.846) |  |
|  | 0–4 | 39,062 | 266 | 1.161 | 0.656 (0.58–0.742) |  |
|  | ≥ 4 | 31,308 | 192 | 1.042 | 0.61 (0.528–0.705) |  |
|  | Current smoker | 548,895 | 5,299 | 1.650 | 1 (Ref.) |  |
| Yes | Non-smoker | 902,467 | 11,047 | 2.094 | 0.497 (0.475–0.521) |  |
|  | Smoking cessation |  |  |  |  |  |
|  | ≤ 0 | 22,128 | 354 | 2.780 | 0.792 (0.71–0.883) |  |
|  | 0–4 | 18,737 | 233 | 2.146 | 0.642 (0.562–0.732) |  |
|  | ≥ 4 | 15,945 | 200 | 2.161 | 0.681 (0.591–0.786) |  |
|  | Current smoker | 219,192 | 4,044 | 3.204 | 1 (Ref.) |  |
| Diabetes |  |  |  |  |  | <0.0001 |
| No | Non-smoker | 2,589,634 | 16,000 | 1.055 | 0.461 (0.444–0.478) |  |
|  | Smoking cessation |  |  |  |  |  |
|  | ≤ 0 | 56,285 | 496 | 1.513 | 0.717 (0.655–0.785) |  |
|  | 0–4 | 51,083 | 403 | 1.347 | 0.662 (0.599–0.732) |  |
|  | ≥ 4 | 42,402 | 320 | 1.286 | 0.651 (0.582–0.729) |  |
|  | Current smoker | 672,928 | 7,385 | 1.879 | 1 (Ref.) |  |
| Yes | Non-smoker | 267,412 | 4,321 | 2.782 | 0.557 (0.52–0.597) |  |
|  | Smoking cessation |  |  |  |  |  |
|  | ≤ 0 | 11,102 | 226 | 3.558 | 0.92 (0.801–1.055) |  |
|  | 0–4 | 6,716 | 96 | 2.480 | 0.647 (0.527–0.794) |  |
|  | ≥ 4 | 4,851 | 72 | 2.570 | 0.704 (0.556–0.891) |  |
|  | Current smoker | 95,159 | 1,958 | 3.596 | 1 (Ref.) |  |
| Hyperlipidemia |  |  |  |  |  | <0.0001 |
| No | Non-smoker | 2,171,641 | 13,130 | 1.033 | 0.483 (0.464–0.502) |  |
|  | Smoking cessation |  |  |  |  |  |
|  | ≤ 0 | 52,818 | 481 | 1.566 | 0.785 (0.716–0.862) |  |
|  | 0–4 | 44,084 | 309 | 1.196 | 0.633 (0.564–0.709) |  |
|  | ≥ 4 | 34,873 | 241 | 1.177 | 0.647 (0.569–0.737) |  |
|  | Current smoker | 616,432 | 6,348 | 1.764 | 1 (Ref.) |  |
| Yes | Non-smoker | 685,405 | 7,191 | 1.794 | 0.469 (0.443–0.497) |  |
|  | Smoking cessation |  |  |  |  |  |
|  | ≤ 0 | 14,569 | 241 | 2.864 | 0.749 (0.657–0.855) |  |
|  | 0–4 | 13,715 | 190 | 2.390 | 0.643 (0.555–0.745) |  |
|  | ≥ 4 | 12,380 | 151 | 2.097 | 0.593 (0.503–0.698) |  |
|  | Current smoker | 151,655 | 2,995 | 3.417 | 1 (Ref.) |  |
| Abdominal obesity |  |  |  |  |  | 0.2418 |
| No | Non-smoker | 2,273,232 | 13,736 | 1.033 | 0.465 (0.448–0.483) |  |
|  | Smoking cessation |  |  |  |  |  |
|  | ≤ 0 | 53,753 | 539 | 1.725 | 0.787 (0.721–0.859) |  |
|  | 0–4 | 42,877 | 331 | 1.318 | 0.641 (0.574–0.716) |  |
|  | ≥ 4 | 30,761 | 242 | 1.340 | 0.693 (0.61–0.788) |  |
|  | Current smoker | 606,921 | 6,907 | 1.951 | 1 (Ref.) |  |
| Yes | Non-smoker | 583,814 | 6,585 | 1.924 | 0.503 (0.473–0.534) |  |
|  | Smoking cessation |  |  |  |  |  |
|  | ≤ 0 | 13,634 | 183 | 2.319 | 0.777 (0.669–0.903) |  |
|  | 0–4 | 14,922 | 168 | 1.937 | 0.682 (0.583–0.797) |  |
|  | ≥ 4 | 16,492 | 150 | 1.560 | 0.585 (0.496–0.69) |  |
|  | Current smoker | 161,166 | 2,436 | 2.607 | 1 (Ref.) |  |

Ischemic stroke

| Subgroup | Smoking status  (weight change) | Number of subjects | Events | IR (per 1000 person years) | HR^a^ (95% Cl) | p-Interaction |
| --- | --- | --- | --- | --- | --- | --- |
| Sex |  |  |  |  |  | 0.8415 |
| Male | Non-smoker | 704,700 | 9,665 | 2.352 | 0.574 (0.558–0.591) |  |
|  | Smoking cessation |  |  |  |  |  |
|  | ≤ 0 | 65,508 | 889 | 2.338 | 0.774 (0.723–0.829) |  |
|  | 0–4 | 56,358 | 598 | 1.816 | 0.648 (0.596–0.703) |  |
|  | ≥ 4 | 46,241 | 464 | 1.713 | 0.655 (0.596–0.718) |  |
|  | Current smoker | 744,941 | 11,225 | 2.590 | 1 (Ref.) |  |
| Female | Non-smoker | 2,152,346 | 23,422 | 1.862 | 0.56 (0.512–0.612) |  |
|  | Smoking cessation |  |  |  |  |  |
|  | ≤ 0 | 1,879 | 26 | 2.400 | 0.738 (0.498–1.095) |  |
|  | 0–4 | 1,441 | 19 | 2.273 | 0.788 (0.498–1.245) |  |
|  | ≥ 4 | 1,012 | 9 | 1.536 | 0.505 (0.261–0.977) |  |
|  | Current smoker | 23,146 | 494 | 3.689 | 1 (Ref.) |  |
| Age |  |  |  |  |  | 0.1674 |
| Age < 55 | Non-smoker | 1,487,778 | 5,119 | 0.588 | 0.546 (0.515–0.579) |  |
|  | Smoking cessation |  |  |  |  |  |
|  | ≤ 0 | 42,089 | 234 | 0.946 | 0.787 (0.69–0.899) |  |
|  | 0–4 | 38,270 | 169 | 0.750 | 0.61 (0.523–0.712) |  |
|  | ≥ 4 | 33,057 | 129 | 0.662 | 0.534 (0.448–0.636) |  |
|  | Current smoker | 533,232 | 3,607 | 1.152 | 1(Ref.) |  |
| Age ≥ 55 | Non-smoker | 1,369,268 | 27,968 | 3.502 | 0.573 (0.556–0.591) |  |
|  | Smoking cessation |  |  |  |  |  |
|  | ≤ 0 | 25,298 | 681 | 4.738 | 0.765 (0.707–0.827) |  |
|  | 0–4 | 19,529 | 448 | 3.995 | 0.664 (0.603–0.73) |  |
|  | ≥ 4 | 14,196 | 344 | 4.208 | 0.701 (0.629–0.781) |  |
|  | Current smoker | 234,855 | 8,112 | 6.067 | 1 (Ref.) |  |
| Hypertension |  |  |  |  |  | 0.9309 |
| No | Non-smoker | 1,954,579 | 13,657 | 1.194 | 0.529 (0.509–0.551) |  |
|  | Smoking cessation |  |  |  |  |  |
|  | ≤ 0 | 45,259 | 407 | 1.541 | 0.74 (0.67–0.819) |  |
|  | 0–4 | 39,062 | 290 | 1.265 | 0.656 (0.583–0.739) |  |
|  | ≥ 4 | 31,308 | 211 | 1.145 | 0.641 (0.559–0.735) |  |
|  | Current smoker | 548,895 | 5,904 | 1.839 | 1 (Ref.) |  |
| Yes | Non-smoker | 902,467 | 19,430 | 3.698 | 0.587 (0.566–0.609) |  |
|  | Smoking cessation |  |  |  |  |  |
|  | ≤ 0 | 22,128 | 508 | 4.000 | 0.777 (0.709–0.851) |  |
|  | 0–4 | 18,737 | 327 | 3.016 | 0.633 (0.566–0.707) |  |
|  | ≥ 4 | 15,945 | 262 | 2.834 | 0.644 (0.569–0.729) |  |
|  | Current smoker | 219,192 | 5,815 | 4.622 | 1 (Ref.) |  |
| Diabetes |  |  |  |  |  | 0.0995 |
| No | Non-smoker | 2,589,634 | 25,480 | 1.682 | 0.562 (0.545–0.58) |  |
|  | Smoking cessation |  |  |  |  |  |
|  | ≤ 0 | 56,285 | 648 | 1.978 | 0.762 (0.703–0.825) |  |
|  | 0–4 | 51,083 | 470 | 1.572 | 0.653 (0.595–0.717) |  |
|  | ≥ 4 | 42,402 | 345 | 1.387 | 0.615 (0.552–0.685) |  |
|  | Current smoker | 672,928 | 8,860 | 2.257 | 1 (Ref.) |  |
| Yes | Non-smoker | 267,412 | 7,607 | 4.924 | 0.623 (0.591–0.658) |  |
|  | Smoking cessation |  |  |  |  |  |
|  | ≤ 0 | 11,102 | 267 | 4.211 | 0.734 (0.648–0.833) |  |
|  | 0–4 | 6,716 | 147 | 3.806 | 0.675 (0.572–0.797) |  |
|  | ≥ 4 | 4,851 | 128 | 4.593 | 0.866 (0.725–1.034) |  |
|  | Current smoker | 95,159 | 2,859 | 5.273 | 1 (Ref.) |  |
| Hyperlipidemia |  |  |  |  |  | <0.0001 |
| No | Non-smoker | 2,171,641 | 22,284 | 1.756 | 0.565 (0.548–0.583) |  |
|  | Smoking cessation |  |  |  |  |  |
|  | ≤ 0 | 52,818 | 634 | 2.066 | 0.74 (0.682–0.802) |  |
|  | 0–4 | 44,084 | 421 | 1.631 | 0.637 (0.577–0.702) |  |
|  | ≥ 4 | 34,873 | 307 | 1.500 | 0.624 (0.557–0.7) |  |
|  | Current smoker | 616,432 | 8,705 | 2.424 | 1 (Ref.) |  |
| Yes | Non-smoker | 685,405 | 10,803 | 2.703 | 0.578 (0.549–0.61) |  |
|  | Smoking cessation |  |  |  |  |  |
|  | ≤ 0 | 14,569 | 281 | 3.341 | 0.834 (0.738–0.943) |  |
|  | 0–4 | 13,715 | 196 | 2.465 | 0.65 (0.562–0.751) |  |
|  | ≥ 4 | 12,380 | 166 | 2.306 | 0.663 (0.567–0.775) |  |
|  | Current smoker | 151,655 | 3,014 | 3.438 | 1 (Ref.) |  |
| Abdominal obesity |  |  |  |  |  | 0.6526 |
| No | Non-smoker | 2,273,232 | 22,215 | 1.673 | 0.561 (0.543–0.579) |  |
|  | Smoking cessation |  |  |  |  |  |
|  | ≤ 0 | 53,753 | 678 | 2.171 | 0.767 (0.709–0.829) |  |
|  | 0–4 | 42,877 | 418 | 1.666 | 0.648 (0.587–0.715) |  |
|  | ≥ 4 | 30,761 | 279 | 1.545 | 0.657 (0.583–0.74) |  |
|  | Current smoker | 606,921 | 8,774 | 2.482 | 1 (Ref.) |  |
| Yes | Non-smoker | 583,814 | 10,872 | 3.187 | 0.591 (0.562–0.623) |  |
|  | Smoking cessation |  |  |  |  |  |
|  | ≤ 0 | 13,634 | 237 | 3.009 | 0.79 (0.692–0.902) |  |
|  | 0–4 | 14,922 | 199 | 2.295 | 0.658 (0.57–0.76) |  |
|  | ≥ 4 | 16,492 | 194 | 2.019 | 0.639 (0.552–0.739) |  |
|  | Current smoker | 161,166 | 2,945 | 3.155 | 1 (Ref.) |  |

IR, incidence rate; HR, hazard ratio; CI, confidence intervals.

^a^Adjusted for age, sex, body mass index, alcohol drinking, low income, and regular exercise.
