# Supplementary material for: Protective effect of smoking cessation on subsequent myocardial infarction and ischemic stroke independent of weight gain: A nationwide cohort study
Source: PLoS One. 2020 Jul 16;15(7):e0235276. doi: 10.1371/journal.pone.0235276 (PMC7365437; doi:10.1371/journal.pone.0235276)
Supplement: Attachment — Submitted filename: Response to Reviewers.docx [file pone.0235276.s003.docx]

**Response to Reviewers**

Dear Editor of PLos ONE,

Thank you for giving us the opportunity to revise our manuscript.

The comments of the reviewers were somewhat difficult to answer, but we tried our best to cope with the comments of the reviewers. In particular, we understood that both reviewers thought the analyses of our study were inappropriate. We took this as much as possible and revised our analyses. Also, we tried to correct the English grammar of our paper to improve communication.

Here are the responses to the comments of the reviewers:

To reviewer #1

1. While the scale of this analysis which includes 5.2 million Korean adults is impressive neither the topic nor the results are novel.

: Thank you for your comment. Although there have been various studies before our study on the relationship between weight gain after smoking cessation and cardiovascular disease, we demonstrated significant results by evaluating the occurrence of real cardiovascular events as outcome in millions of people including both sexes across the country, complementing the shortcomings of these studies.

2. The analytic approach is confusing and potentially flawed. The dependt variable of a 1-2 point change in BMI appears to be differential depending on an individual's height. For example, a 6 foot 1 inch individual demonstrating an 8 pound weight gain from 200 to 208 pounds would show a 1.0 change in BMI (from 26.4 to 27.4). Conversely a 5 foot 6 inch individual at a baseline weight of 120 pounds which show a 4.5 pound weight gain in order to achieve a 1.0 change in BMI (from 19.1 to 20.1). A much more straightforward approach to the analyses would have simply reported an overall change in weight rather than BMI.

: Thank you for your comment. Based on your feedback, we’ve conducted a new analysis with a straightforward approach using weight, not BMI. As a result, it is consistently confirmed that weight gain after smoking cessation does not adversely affect the cardiovascular protection effect.

3. Introduction: The text is very general and seems to overlook relative versus absolute effects of cardiac risk factors. The text does not include any information about affect sizes. Among all the risk factors identified smoking far and away is the most significant.

: Thank you for your comment. We also agree that smoking cessation is the most effective and powerful factor for reducing the risk of cardiovascular disease. We’ve revised the text so that the relative risk is not overestimate relative to the absolute risk of smoking.

4. Study population: The exclusion of 17,000 individuals diagnosed with an MI or ischemic stroke within 1 year of follow-up is confusing.

: Thank you for your comment. We initially thought it was unclear whether a casual relationship existed between the incidence of myocardial infarctions or strokes within one year from baseline and exposure to weight change. However, we agree that you were confused with this approach. We’ve included all MIs and ISs that occurred after the baseline without a one-year washout period in the analyses.

5. Classification of the population into smokers and "those who had not smoked during this time" as non-smoker is confusing and inaccurate. Moreover, this approach is inconsistent with the common classification system noting individuals as current, former or never smokers.

: Thank you for your comment. We evaluated the subjects’ smoking history through self-questionnaires during health screenings including their current smoking status, duration, and amount of smoking. The participants responded to their current smoking status through one of three choices: never smoked, smoking in the past but now quitting, or continuing to smoke. Current smokers were defined as those who responded to smoke continuously from 4 years prior to baseline. Those who consistently never smoked were classified as non-smokers. The participants who quit smoking at baseline, but who were smokers 4 years prior were classified as the smoking cessation group. We’ve added these to the text to avoid confusion of communication.

6. table 1. What is "difference" under systolic blood pressure, diastolic blood pressure, fasting plasma glucose, total cholesterol?

: Thank you for your comment. The "difference" of these characteristics refers to the numerical change of the baseline and the 4 year prior. For example, systolic (1.0 ± 16.1 mmHg) and diastolic blood pressure (0.3 ± 11.5 mmHg), blood cholesterol (5.5 ± 34.7 mg/dl), and fasting blood sugar (5.1 ± 26.0 mg/dl) increased in the smoking cessation group at baseline compared to the 4 year prior.

7. Table 2. Body mass index is not weight gain. Adding additional variables into their multivariate models parens alcohol, income, exercise, hypertension, diabetes, hyperlipidemia) does not change risk estimates beyond more parsimonious models. Authors are urged to simplify their models and descriptions.

: Thank you for your comment. In the selection of confounders in our study, we selected variables that influence the cardiovascular outcomes. We agree that chronic disease such as hypertension, diabetes, and hyperlipidemia are not appropriate to be included as variables under the influence of weight gain. We’ve subtracted these mediators from variables, and instead presented a subgroup analysis with or without mediators.

8. Table 3. The results and conclusions would be much easier to understand if they were presented as actual weight gains rather than changes in BMI.

: Thank you for your comment. As we answered in comment #2, we’ve conducted a new analysis using actual weight gains.

9. Supporting information is confusing. Figure 1 raises additional questions about the study design since it suggests that BMI was assessed over an interval of 4 years prior to baseline while MI and ischemic strokes were assessed for a period of 5 years after baseline. Content of figure 2 is also unclear.

: Thank you for your comment. As you said, weight change was assessed over an interval of 4 years prior (2005~2006) to baseline (2009 ~ 2010). MI and ischemic strokes were followed up from baseline (2009 ~ 2010) to the end of 2015, and the mean follow-up periods for MI and ischemic strokes were 5.9 ± 0.7 years and 5.8 ± 0.8 years, respectively. We’ve modified figure 1 and deleted figure 2.

10. As a minor point the manuscript would benefit from careful editing to clarify wording and grammar.

: We thank you for pointing out this and have tried to clarify wording and correct the English grammar to improve communication.

To reviewer #2

1. The first sentence of the second paragraph is confusing. Consider rewording.

: Thank you for your comment. We’ve reworded the first sentence of the second paragraph which can be confusing.

2. Consider rewording the second sentence to "Clair et al. [8] showed that body weight change after smoking cessation....."

: Thank you for your comment. We’ve reworded the second sentence to “In the study using cohort data from the Framingham Offspring Study...”

3. Explain what you mean by "mortality seems better in higher BMI groups"

: Thank you for your comment. Cardiovascular prognosis and mortality seems better in overweight or obese described by the “obesity paradox”. This means that the risk of actual cardiovascular events due to weight gain might be underestimated when determining mortality as an outcome. As a result of the study by Hu Y et al. mentioned in that section, recent quitters that did not gain weight had a higher risk of mortality relative to those who gained weight.

Methods

Study population:

4. Correct the following sentence"Our study approved by the NHIS" to "Our study WAS approved by the NHIS"

: Thank you for your comment. We’ve corrected it as your comment.

5. Correct "4 years ago" to "4 years PRIOR" in line 77/78

: Thank you for your comment. We’ve corrected it as your comment.

6. Correct "Individuals diagnosed with previous MIs or ISs" to "Individuals PREVIOUSLY DIAGNOSED with MIs or ISs"

: Thank you for your comment. We’ve corrected it as your comment.

7. Correct "We further excluded 17,192 patients who diagnosed with MI or IS..." to "We further excluded 17,192 patients who WERE diagnosed with MI or IS..."

: Thank you for your comment. We’ve removed this sentence from the text.

8. Explain why 17,192 who were diagnosed with MI or MS were excluded. What was unclear about the causal relationship?

: Thank you for your comment. We initially thought it was unclear whether a casual relationship existed between the incidence of myocardial infarctions or strokes within one year from baseline and exposure to weight change. However, we agree that you and reviewer #1 were confused with this approach. We’ve included all MIs and ISs that occurred after the baseline without a one-year washout period in the analyses.

9. Excluding patients with the outcome without further explanation contributes to selection bias.

: Thank you for your comment. Since our study made the first diagnosed cardiovascular disease as an outcome, the participants who were previously diagnosed with cardiovascular disease could not be included in the study. In addition, people with a history of cardiovascular disease could have unintentional weight loss and/or sarcopenia. This can be a bias to the weight change after smoking cessation, an exposure of our study.

Definition of smoking history and outcomes:

10. Replace "years ago" with "years prior"

: Thank you for your comment. We replaced it with "years prior".

11. Explain what you mean by "self-questionnaire". Do you mean self-report?

: Thank you for your comment. “Self-questionnaire" are questions that must be answered in a medical examination. Questions on current smoking status, duration, and amount of smoking are written in the questionnaire. The participants responded to their current smoking status through one of three choices: never smoked, smoking in the past but now quitting, or continuing to smoke.

12. How did you define "steadily smoking"? Consider adding the actual questions that were asked to this section. Same for those who quit.

: Thank you for your comment. The participants responded to their current smoking status through one of three choices: never smoked, smoking in the past but now quitting, or continuing to smoke. Current smokers were defined as those who responded to smoke continuously from 4 years prior to baseline. Those who consistently never smoked were classified as non-smokers. The participants who quit smoking at baseline, but who were smokers 4 years prior were classified as the smoking cessation group. We’ve added these to the text to avoid confusion of communication.

13. State the follow-up period in this section.

: Thank you for your comment. We’ve written a follow-up period in this section. The mean follow-up periods for MI and ischemic strokes were 5.9 ± 0.7 years and 5.8 ± 0.8 years, respectively.

14. This sentence in unclear "The exclusion criteria for previous MIs or ISs were the same as above." State clearly what the exclusion criteria were.

: Thank you for your comment. The occurrence of a MI was defined as an ICD-10 I21 or I22 code claimed at least twice, or more than once with a hospitalization. The occurrence of an IS was defined as an ICD-10 I63 or I64 code claimed together with a hospitalization and a radiological examination (magnetic resonance imaging or computed tomography). The participants who had a history of MI or IS identified using these ICD code claims prior to baseline were excluded. We've updated this in the text.

Other baseline characteristics:

15. Include sex and age in this section.

: Thank you for your comment. We’ve written statements about age and sex in this section.

Statistical analysis:

16. It is unclear whether this was a primary or secondary data analysis.

: Thank you for your comment. We’ve corrected the paragraph so that the primary and secondary analyses are not confused.

17. Incidence rates are obtained by a Binomial/Poisson regression, Risk Differences are obtained by a normal/log-normal regression, and hazard ratios are obtained by time-to-even regression. These are all different models assessing different measures. Please explain which procedure was used to obtain which measure and what the exposure and outcome were for each procedure. Also, explain whether or not models were adjusted, and if so, the variables that were adjusted for.

: Thank you for your comment. The incidence rates were expressed as crude rates without statistical test analysis. HRs in primary and secondary analyses were analyzed using a Cox proportional hazards model with a 95% confidence interval (CI). We’ve added a description of variables used for adjustment in each model to the text.

Table 1:

18. Indicate which variables are shown as N(%) and which are shown as Mean (SD).

: Thank you for your comment. We’ve inserted N(%) and Mean(SD) in Table 1, which are appropriate for each characteristics.

19. Add a column fourth for the total and percentages for smoking status.

: Thank you for your comment. We’ve added the number of subjects in each group, along with the percentages of the total.

20. Replace "Number" by "Characteristic" and "No. (%)" by "N (%)".

: Thank you for your comment. The "number" we entered was the number of subjects in each group in the row. We’ve modified "No. (%)" to "N (%)".

21. Footnote: use lower case "p" indicating p-value.

: Thank you for your comment. We replaced uppercase P by lowercase.

22. What do you mean by "All characteristics met P < 0.0001"? What is being being tested here? Please specify.

: Thank you for your comment. This indicates that there are significant differences in characteristics of the three groups according to smoking history. The difference between the three groups is significant, even if only one comparison of the three groups is significant.

23. Add the proportion of daily and non-daily smokers to Table 1. Pack years cannot be calculated for non-daily smokers.

: Thank you for your comment. Unfortunately, the daily and non-daily smokers cannot be distinguished from the questionnaire used in the medical examination, so this may be a limitation of our study. We describe this 'point prevalence' design in the limitation section of the discussion.

Table 2:

24. Consider changing the reference group to current smokers.

: Thank you for your comment. HRs were compared among the smoking cessation, non-smoker, and current-smoker groups with current smokers as a reference group. If the meaning of this comment needs to be modified "1 (Ref.)", we will revise it as soon as you respond.

25. Was the incidence rate adjusted or not?

: Thank you for your comment. The incidence rates were expressed as crude rates without adjustment.

Table 3:

26. Consider changing the reference group to current smokers.

: Thank you for your comment. HRs were compared among the smoking cessation, non-smoker, and current-smoker groups with current smokers as a reference group. If the meaning of this comment needs to be modified "1 (Ref.)", we will revise it as soon as you respond.

27. Was the incidence rate adjusted or not?

: Thank you for your comment. The incidence rates were expressed as crude rates without adjustment.

28. It would be worthwhile to perform the regression of the outcomes on BMI (4 categories) stratified by smoking status adjusting for all identified confounders except BMI.

: Thank you for your comment. We agreed with reviewer # 1's comment #2, so we've conducted a new analysis with a straightforward approach using weight, not BMI. Therefore, baseline BMI has been included in variables.

Results

29. Please do not use risk ratio and hazard ratio interchangeably, RR does not take into effect the time to event while HR does.

: Thank you for your comment. We have fixed the risk ratio to the hazard ratio as your opinion.

Discussion

30. Be aware of overarching statements or statements that are not supported by evidence such as the first 2 sentences of the discussion.

: Thank you for your comment. We’ve tried to reduce exaggerated expressions that are not supported by evidence.

31. This paper did not assess the effect of BMI on the risk of MS and IS as mentioned in the second paragraph of the discussion, however, this would be possible with the analyses that I previously suggested under table 3.

: Thank you for your comment. As we responded to your comment #28, we’ve conducted a new analysis using weight.

32. Discussion will likely have to be revised according to the suggested analyses.

: Thank you for your comment. We’ve added related contents to the discussion.

General:

33. Consider restricting the analysis to those who are 40 years of age or older as incidence of cardiovascular outcomes are likely to be low in the younger age groups.

: Thank you for your comment. We agree with your comment and have restricted the research group to those over 40 years of age. The results are consistent after restriction.

34. Specify the novelty of this article and what value it adds to the existing literature.

: Thank you for your comment. Although there have been various studies before our study on the relationship between weight gain after smoking cessation and cardiovascular disease, we demonstrated significant results by evaluating the occurrence of real cardiovascular events as outcome in millions of people including both sexes across the country, complementing the shortcomings of these studies.

35. Figure 2 does not add any more information than Table 3, consider removing.

: Thank you for your comment. We’ve retained Table 3 and removed figure 2.

We personally thank the reviewers for spending their precious time to read and suggest comments for our manuscript.

We hope that our revision satisfy the reviewers and could be accepted this time.

Thank you again for your dedication to PLos ONE.

Best regards,

Won-Young Lee, MD, PhD

Division of Endocrinology and Metabolism, Department of Internal Medicine, Kangbuk Samsung Hospital, Sungkyunkwan University School of Medicine, 29 Saemunan-ro, Jongno-gu, Seoul 03181, Republic of Korea

Tel: +82-2-2001-2579; E-mail: [drlwy@hanmail.net](mailto:drlwy@hanmail.net)

Eun-Jung Rhee, MD, PhD

Division of Endocrinology and Metabolism, Department of Internal Medicine, Kangbuk Samsung Hospital, Sungkyunkwan University School of Medicine, 29 Saemunan-ro, Jongno-gu, Seoul 03181, Republic of Korea

Tel: +82-2-2001-2579; E-mail: hongsiri@hanmail.net
